# AtMYB50 regulates root cell elongation by upregulating *PECTIN METHYLESTERASE INHIBITOR 8* in *Arabidopsis thaliana*

**DOI:** 10.1371/journal.pone.0285241

**Published:** 2023-12-22

**Authors:** Kosuke Mase, Honomi Mizuno, Norihito Nakamichi, Takamasa Suzuki, Takaaki Kojima, Sho Kamiya, Taiga Takeuchi, Chiko Kondo, Harumi Yamashita, Satomi Sakaoka, Atsushi Morikami, Hironaka Tsukagoshi

**Affiliations:** 1 Faculty of Agriculture, Meijo University, Nagoya, Aichi, Japan; 2 Graduate School of Bioagricultural Sciences, Nagoya University, Nagoya, Aichi, Japan; 3 Department of Biological Chemistry, College of Bioscience and Biotechnology, Chubu University, Kasugai, Aichi, Japan; University of Massachusetts Amherst, UNITED STATES

## Abstract

Plant root development involves multiple signal transduction pathways. Notably, phytohormones like auxin and cytokinin are well characterized for their molecular mechanisms of action. Reactive oxygen species (ROS) serve as crucial signaling molecules in controlling root development. The transcription factor, *UPBEAT1* (*UPB1*) is responsible for maintaining ROS homeostasis at the root tip, influencing the transition from cell proliferation to differentiation. While UPB1 directly regulates peroxidase expression to control ROS homeostasis, it targets genes other than peroxidases, suggesting its involvement in root growth through non-ROS signals. Our investigation focused on the transcription factor *MYB50*, a direct target of UPB1, in *Arabidopsis thaliana*. By analyzing multiple fluorescent proteins and conducting RNA-seq and ChIP-seq, we unraveled a step in the MYB50 regulatory gene network. This analysis, in conjunction with the *UPB1* regulatory network, demonstrated that MYB50 directly regulates the expression of *PECTIN METHYLESTERASE INHIBITOR 8* (*PMEI8*). Overexpressing *PMEI8*, similar to the *MYB50*, resulted in reduced mature cell length. These findings establish MYB50 as a regulator of root growth within the UPB1 gene regulatory network. Our study presents a model involving transcriptional regulation by MYB50 in the UPB1 regulated root growth system and sheds light on cell elongation via pectin modification.

## Introduction

Plant roots are important organs that support the entire plant body, absorb water and nutrients from the soil, and respond to environmental changes. To achieve optimal root architecture, the root developmental mechanism is strictly controlled. Maintaining the balance of transition from cell proliferation to differentiation at the root tip is a key to coordinating primary root growth. Cell proliferation occurs in the meristematic zone that includes the stem cell niche. Once the cell leaves the meristematic zone, it stops proliferating but starts rapidly elongating in the elongation zone. After the cells fully elongate, the cells mature into their final states and function in the maturation zone. Maintaining the precise balance of cell status among these zones is vital for root growth. For this process, the transcriptional network is important [1, 2] and almost all hormones are involved in the transcriptional network for regulating the balance between cell proliferation and differentiation at the root tips [3, 4].

In addition to hormones, reactive oxygen species (ROS) also act as regulators to maintain the balance of cell function in the root tip. ROS, such as superoxide, hydrogen peroxide, and hydroxyl radicals, are thought of as molecules that are harmful to the cells as they are highly reactive. However, roots accumulate ROS at some level to control root growth [5]. Hydrogen peroxide represses the expression of cell cycle-related genes in the meristematic zone [6]. *UPBEAT1* (*UPB1*), a bHLH domain-containing transcription factor, is a key regulator of the balance between cell proliferation and elongation by regulating the expression of several peroxidase genes. The spatial accumulation patterns of superoxide and hydrogen peroxide influence the size of the meristematic and elongation zones [7]. *MYB30* is an ROS-responsive transcription factor and *MYB30* regulates root cell elongation under ROS and abscisic acid signaling [8, 9]. ANAC032 regulates *MYB30* expression and a transcription network that modulates cell elongation [10]. These results indicate that ROS regulates both cell proliferation and elongation in the root. Interestingly, *MYB30* and *ANAC032* are not direct targets of UPB1.

In the present study, we focused on the UPB1 gene regulatory network, which regulates primary root growth. Although UPB1 directly regulates the expression of several peroxidase genes, it also targets other genes. 2,375 UPB1-responsive genes are identified by microarray analysis and 166 putative UPB1 direct targets are identified by ChIP-chip analysis [7]. To elucidate UPB1 regulatory transcriptional network, we focused on the transcription factors among the 166 UPB1 direct targets. Among them, we chose a transcription factor, *MYB50* whose function had not yet been analyzed. Functional analysis of MYB50 can provide insights into root growth regulation under the UPB1 gene regulatory network. We confirmed the transcriptional network from *UPB1* to *MYB50* by multi-color time-lapse imaging of *A*. *thaliana* roots. We also investigated MYB50 regulatory networks using RNA-seq and ChIP-seq analyses. Based on these analyses, we found that MYB50 directly regulated the expression of several cell wall modification genes. We further investigated the root phenotypes of the overexpressor of MYB50 target genes, especially *PECTIN METHYLESTERASE INHIBITOR 8* (*PMEI8*). *PMEI8* overexpression resulted in a shorter mature cell length in the root. These results indicate that UPB1 regulates root growth through *MYB50* expression as a transcription factor for controlling further downstream gene expression and that MYB50 regulates cell elongation by controlling cell wall modification genes under the UPB1 transcription network.

## Material and methods

### Plant materials and growth conditions

*Arabidopsis thaliana* Columbia-0 (Col-0) was used as the wild-type. The T-DNA insertion line, *upb1-1* was obtained from the SLAK collection and the seed stock center of the Arabidopsis Biological Resource Center (ABRC). *upb1-1* mutant was genotyped using a left-border primer on T-DNA (LB), right-side primers on the genome (RP), and left-border primers on the genome (LP) as described previously [7].

All seeds were sterilized with 1% bleach and 0.05% Triton X-100 for 5 min and then washed thrice with sterilized water. Seeds were germinated on Murashige and Skoog (MS; FUJIFILM Wako Pure Chemical, Osaka, Japan) medium supplemented with 1% sucrose and 1% agar after two days at 4˚C. Plants were grown vertically in a chamber (Panasonic, Osaka, Japan) at 22˚C with 16-hour-light/8-hour-dark cycle. For estradiol treatment, 6-day-old seedlings were transferred onto MS agar plates containing 5 μM estradiol (FUJIFILM Wako Pure Chemical).

### Plasmid construction and plant transformation

Genomic DNA from Columbia was used as a template for the amplification of the 3,020 bp upstream region of *pMYB50* for promoter cloning. One base of 5’ dA overhang was added to the PCR amplicon of *pMYB50* using Taq polymerase (Takara Bio Inc., Shiga, Japan), which was then cloned into pENTR5’-TOPO (Thermo Fischer Scientific, Waltham, MA, USA) and named pENTR5’-*pMYB50*. For the genomic coding region and cDNA for *MYB50* and *PMEI8* cloning, *MYB50* genomic and cDNA regions and the cDNA region of *PMEI8* were amplified using the forward primer containing CACC sequences for TOPO cloning and the reverse primer. *CACC-gMYB50*, *CACC-cMYB50*, and *CACC-cPMEI8* fragments were cloned into pENTR/D-TOPO (Thermo Fischer Scientific). For *YFP-cMYB50*, *YFP-cPMEI8*, *YFP-cUPB1*, and *CFP-cUPB1* cloning, the *cMYB50*, *cPMEI8*, and *cUPB1* cDNA regions were amplified using forward and reverse primers containing the BamHI site just before the *MYB50*, *PMEI8*, or *UPB1* termination codon. The *cMYB50*-Bam HI, *cPMEI8*-Bam HI, and *cUPB1*-Bam HI fragments were cloned into Aor51H1, and the Bam HI sites of *YFP*-Aor51H1-Bam HI-pDONR201 plasmids [8] and *CFP*-Aor51H1-Bam HI-pDONR201 plasmids [11], respectively.

For the *pMYB50*::*gMYB50-GFP* construct, pENTR5’-*pMYB50* and *gMYB50* containing pENTR/D-TOPO were cloned into R4pGWB650 [12] using LR Clonase II (Thermo Fischer Scientific). For the *pXVE*::*YFP-cMYB50*, *pXVE*::*YFP-cPMEI8*, *pXVE*::*YFP-cUPB1*, and *pXVE*::*CFP-cUPB1* constructs, *YFP-cMYB50*, *YFP-cPMEI8*, *YFP-cUPB1*, and *CFP-cUPB1* containing pDONR201s were cloned into pMDC7 [13] using LR clonase II.

The resulting plasmids (*pMYB50*::*gMYB50-GFP*, *pXVE*::*YFP-cMYB50*, *pXVE*::*YFP-cPMEI8*, *pXVE*::*YFP-cUPB1*, and *pXVE*::*CFP-cUPB1*) were transformed into *Agrobacterium tumefaciens* (C58C1 pMP90) cells and transformed into Columbia and *upb1-1* mutants. For the *pXVE*::*CFP-cUPB1*/*pMYB50*::*gMYB50-GFP* double-reporter line, *pXVE*::*CFP-cUPB1* containing *Agrobacterium tumefaciens* (C58C1 pMP90) cells were transformed into *pMYB50*::*gMYB50-GFP* plants. The sequences of primers used in this study are provided in S1 Table.

### Quantitative real-time RT-PCR

RNA was isolated from the roots by using the RNeasy Plant Kit (QIAGEN, Hilden, Germany). For RNA isolation from meristematic and elongation zones, 6-day-old plants were dissected as previously described [7]. First-strand cDNA was synthesized using ReverTra Ace qPCR RT Master Mix with gDNA Remover (TOYOBO Co., Ltd., Osaka, Japan). Quantitative real-time RT-PCR (RT-qPCR) was performed using THUNDERBIRD SYBR qPCR Mix (TOYOBO) on a real-time PCR Eco system (PCRmax, Stone Staffordshire, UK). The sequences of primers used in this study are provided in S1 Table. The RT-qPCR efficiency and CT values were determined using standard curves for each primer set. The efficiency-corrected transcript values of three biological replicates for all samples were used to determine the relative expression values. Each value was normalized against the level of *PDF2* [14].

### RNA-seq experiments

Total RNA was isolated from roots; cDNA libraries were generated from 500 ng of total RNA using the NEBnext Ultra II RNA Library Prep kit (New England Biolabs, Ipswich, MA, USA), following the manufacturer’s protocols. The ends of the cDNA libraries were sequenced for 60 cycles using a paired-end module on the Illumina NextSeq 500 platform (Illumina, San Diego, CA, USA). Two biological replicates were used in each experiment.

### RNA-seq data analysis

Short-read sequencing results were mapped to the *A*. *thaliana* genome (TAIR10: www.arabidopsis.org/) using the Bowtie software [15]. These datasets were normalized, and the false discovery rate (FDR) and fold change were calculated using the edgeR package for R [16]. We used an FDR of *q* < 0.001 as the cut-off to determine differentially expressed genes. Data were deposited in the DNA Data Bank of Japan (DDBJ) sequence Read Archive (DRA) (https://www.ddbj.nig.ac.jp/index-e.html) under accession number DRA016078.

### ChIP-seq experiments

Over 1,200 plants per genotype were grown on MS medium for six days. These plants were then transferred onto MS medium containing 5 μM estradiol. One day after transfer, whole roots were fixed and flash-frozen using liquid nitrogen according to a previously described protocol [7]. ChIP was performed as described previously [17] with anti-GFP antibody (ab290, Abcam, Cambridge, UK). ChIP DNA library was generated with a ChIP-Seq Sample Prep Kit (Illumina, San Diego, CA, USA) and the resulting DNA library was analyzed using GAII (Illumina) as described previously [17].

### ChIP-seq data analysis

Base calling of sequence reads obtained by GAII was performed using the GAII pipeline software. Mapping of these sequence reads was performed using Bowtie [15] with default parameters. The resulting sequence alignment/map (SAM) file was converted into a binary alignment/ map (BAM) format file by Samtools 0.1.18 [18]. Significant ChIP DNA peaks (FDR *q* < 10^−20^) were annotated as YFP-MYB50 binding loci using the Model-based Analysis of ChIP-Seq (MACS2) software [19], with the genome size parameter dm (1.2e^8^). The forward- and reverse-peak distributions were validated using MACS2 and visualized using R (http://www.R-project.org/). BAM and indexed BAM files were used to visualize mapping patterns using Integrative Genomics Viewer 2.13.0. Based on the sequence data of 1,000 bp upstream from the translation start site for each gene, obtained from the TAIR database (TAIR10), genes near each peak were identified using a local BLAST tool [20] with the following parameters: blastn -evalue 0.1 -outfmt 6. Manual curation was performed to remove incorrect annotations arising from similarities in promoter sequence regions. The ChIP-seq data were deposited in the DNA Data Bank of Japan (DDBJ) sequence Read Archive (DRA) (https://www.ddbj.nig.ac.jp/index-e.html) under accession number DRA016088.

### Phenotyping and microscopy

To measure the whole root length, the roots were scanned using a flatbed scanner GT-7400U (Epson, Nagano, Japan) while growing on plates. The root length was measured by importing the scanned images into Fiji (imagej.net/software/fiji/). Prior to confocal imaging, roots were stained with propidium iodide (PI; FUJIFILM Wako pure chemical; 10 μg mL^-1^ in water) for 3 to 5 min. Confocal microscopy was performed using a Leica SP8 system (Leica Camera AG, Wetzlar, Germany) with 448 nm excitation and 460–510 nm emission for CFP, 488 nm excitation and 500–550 nm emission for GFP, 488 nm excitation and 490–543 nm emission for YFP, and 555 nm excitation and 580–680 nm emission for PI. Image assembly was performed using the LAS X software (Leica Camera AG).

For time-lapse imaging, Lab-Tek Chambered Coverglass w/cvr (Thermo Fisher Scientific) was used, as described previously [8]. For time-lapse imaging of multi-color reporter line, 6-day-old seedlings of *pXVE*::*CFP-cUPB1*/*pMYB50*::*gMYB50-GFP/upb1-1* in MS medium were placed on chambered cover glass in media containing 5 μM estradiol. The chambers containing plants were imaged using a Leica SP8 confocal microscope at objectives of 20×. Time-lapse images were captured using LAS X every 40 min for 7 h and 20 min. Images were also assembled with LAS X. To quantify CFP-cUPB1 and gMYB50-GFP intensities, 50 z-stack images of the roots were taken with LAS X every 20 min for 5 h using 40× (oil) objectives. Of the 50 z-stack images for each time point, the image with the sharpest focus on the nucleus of the cortex or epidermal cells was selected, and CFP and GFP intensities were quantified as a mean intensity using Fiji software.

To measure the increase in root length, 4-day-old seedlings in MS medium were placed on chambered cover glass in media containing 5 μM estradiol. The chambers containing the plants were imaged using a DMI 6000 B-AFC fluorescence microscope (Leica Camera AG) under a 20× objective lens. Time-lapse images were captured every 30 min for 24 h. The increase in root length was measured using the Fiji software on a time series consisting of images taken every 30 min.

### Statistical analysis

All statistical analyses were performed using Microsoft Excel or R. Hypergeometric testing for overlapping genes between ChIP and RNA-seq was performed using the online program http://nemates.org/MA/progs/overlap_stats.html. Details of the analyses are provided in the figure legends.

## Results

### UPB1 regulates *MYB50* expression at the elongation zone

**Fig 1:**
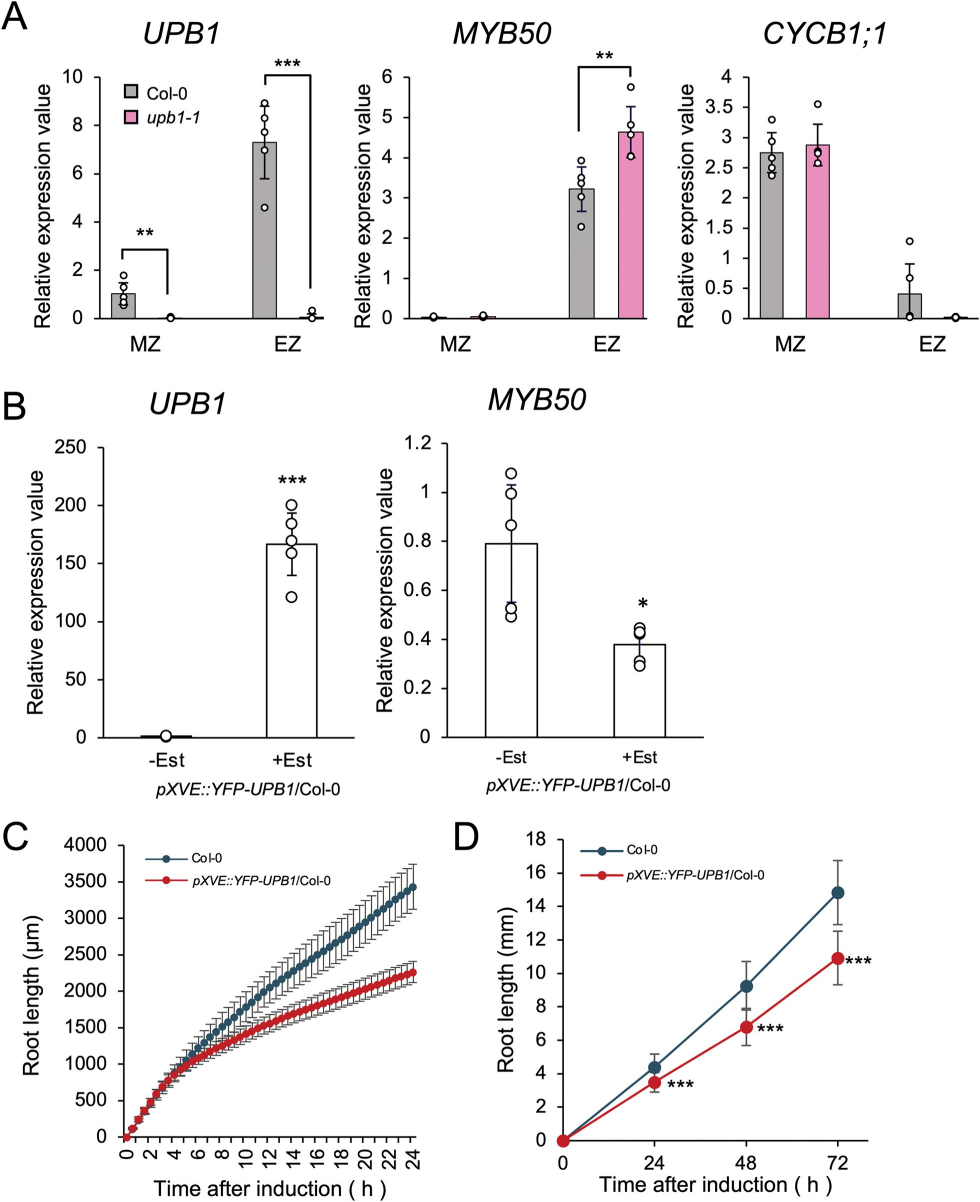
UPB1 regulates *MYB50* expression at the elongation zone. (A) *UPB1*, *MYB50*, and *CYCB1;1* expression in both of the meristematic and elongation zones in roots of 7-day old seedlings of Columbia (Col-0; gray) and *upb1-1* (pink) as measured by RT-qPCR (n = 5). MZ and EZ indicate the meristematic and elongation zone, respectively. Bars show mean ± SD. Statistically significant differences were determined using Student’s *t*-test by comparing Columbia and *upb1-1* in each zone (*** *p* < 0.001; ** *p* < 0.01). *p* value: comparison of *UPB1* expression in MZ of Columbia and *upb1-1*, *p* = 0.002; comparison of *UPB1* expression in EZ of Columbia and *upb1-1*, *p* < 0.001; comparison of *MYB50* expression in EZ of Columbia and *upb1-1*, *p* = 0.0096. (B) *UPB1* and *MYB50* expression analysis of roots of 6-day old seedlings in *pXVE*::*YFP-UPB1*/Columbia with and without 5 μM estradiol (Est) treatment for 24 h measured by RT-qPCR (n = 5). Statistically significant differences were determined using Student’s *t*-test compared with and without estradiol treatment (*** *p* < 0.001; * *p* < 0.05). *p* value: comparison of *UPB1* expression, *p* < 0.001; comparison of *MYB50* expression, *p* = 0.016. (C) Root length in 4-day-old seedlings treated with 5 μM estradiol within the agar medium. Root length was measured every 30 min using time-lapse imaging (n = 5, means ± SD). (D) Root length in 4-day-old seedlings grown vertically on agar medium containing 5 μM estradiol. Root length was measured every 24 h for 72 h (Columbia, n = 38; *pXVE*::*YFP-UPB1*, n = 25, means ± SD). To assess statistical significance, we employed Student’s *t*-test for comparisons between Columbia and transgenic at each time (*** *p* < 0.001).

To confirm previous results [7], we analyzed *MYB50* expression in the *upb1-1* mutant by RT-qPCR by using RNA extracted separately from meristematic and elongation zones. Proper dissection was confirmed by assessing the expression of zone-specific genes *CYCB1;1* and *UPB1*, which are predominantly expressed in the meristematic and elongation zones, respectively (Fig 1A). *MYB50* expression was significantly higher in the elongation zone of the *upb1-1* mutant than in Col-0 plants (Fig 1A). Consistent with previous results [7], UPB1 weakly suppressed *MYB50* expression in the elongation zone. We constructed an estradiol-inducible *YFP-UPB1* transformant (*pXVE*::*YFP -UPB1*). Following 24 h of 5 μM estradiol treatment, *UPB1* expression substantially increased, resulting in a statistically significant 50% reduction in *MYB50* expression (Fig 1B). Time-lapse imaging was performed every 30 min after estradiol treatment for 24 h to measure *pXVE*::*YFP-UPB1* root elongation. After estradiol treatment, elongation of *pXVE*::*YFP-UPB1* roots was inhibited by about a factor of two compared to Columbia (Fig 1C). In addition to time-lapse imaging, we measured the root length of *UPB1* overexpressor on vertical plates containing 5 μM estradiol at 24, 48, and 72 h (Fig 1D). After 72 h of estradiol treatment, the increase in root length in *UPB1* overexpressor was reduced to approximately 70% of that in the Columbia. These findings indicate that *UPB1* overexpression repressed *MYB50* and led to a reduction in root growth, similar to previous results [7].

**Fig 2:**
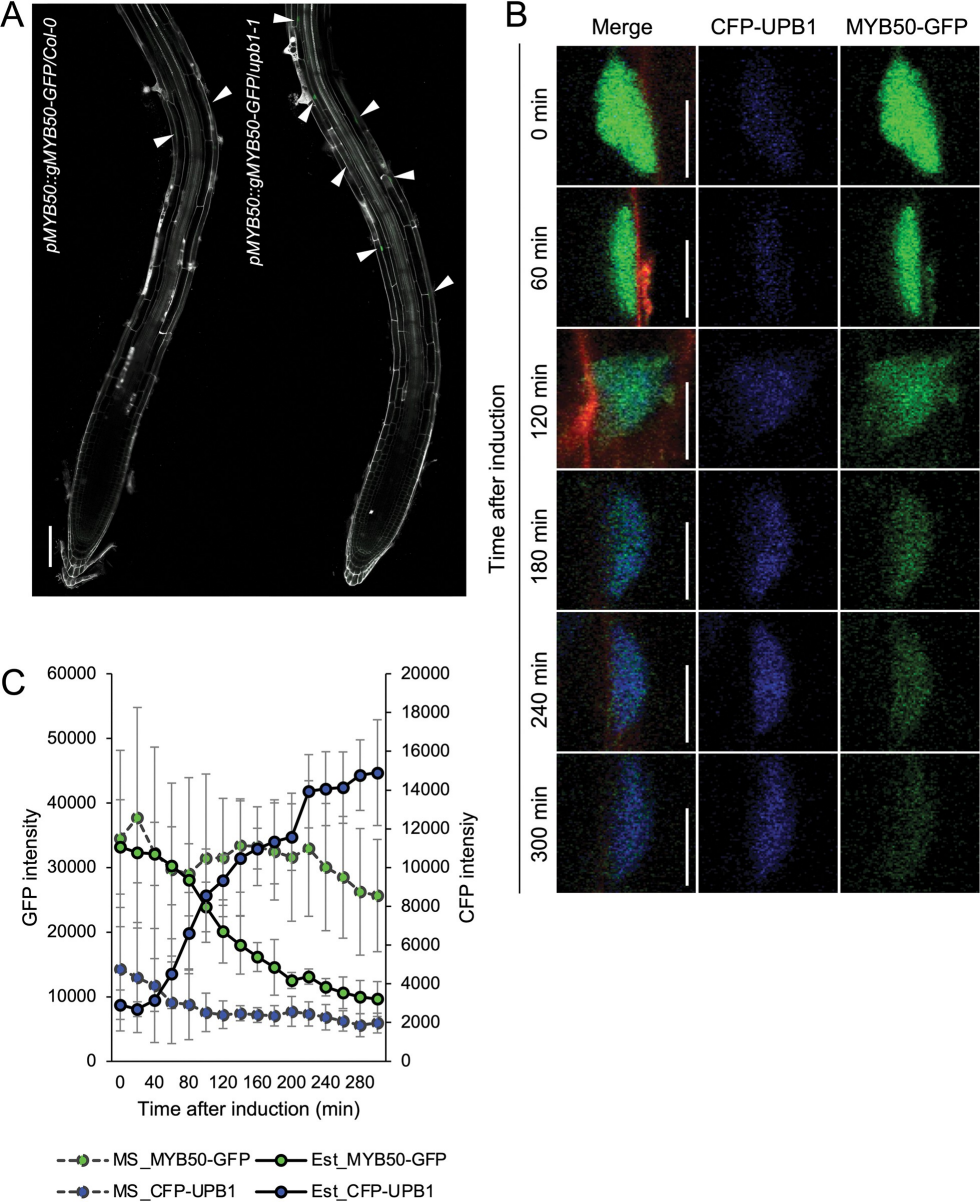
*MYB50* expression pattern in the primary root and the multi-color fluorescent protein expression of CFP-UPB1 and MYB50-GFP. (A) Confocal microscopic images of primary root tip of 7-day old seedlings. White arrow heads indicate the nuclei with apparent GFP fluorescence. Scale bars, 100 μm. (B) Timepoint images every 60 min in the visualization of transcriptional repression of MYB50 by UPB1 in one nucleus. 6-day-old seedlings were stained by propidium iodide (PI) and CFP-UPB1 and each MYB50-GFP signal was independently detected. Apparent nuclei exhibiting detectable CFP or GFP fluorescence were visualized and enlarged. Left panels: merged images of CFP, GFP, and PI, Middle panels: CFP channel, Right panels: GFP channels. Scale bars, 5 μm. (C) Quantification of CFP-UPB1 and MYB50-GFP intensity after estradiol treatment (solid lines) and no-estradiol treatment (dashed lines) every 20 min. Each signal intensity was measured every 20 min using time-lapse imaging (n = 4: two nuclei from each of the two roots, means ± SD).

We examined *MYB50* expression patterns in roots by creating a translational fusion reporter (*pMYB50*::*gMYB50-GFP*) expressed in both in Columbia and *upb1-1*. In Columbia, we observed MYB50-GFP fluorescence in the early differentiation zone but not in the meristematic and the beginning of the apparent elongation zone (Fig 2A and S1 Fig). However, in the *upb1-1* mutant, weak MYB50-GFP fluorescence emerged from the middle of the apparent elongation zone. In both backgrounds, towards the mature region of the root, we noticed fluorescence appeared in large puncta, probably corresponding to nuclei (Fig 2A and S1 Fig), consistent with UPB1 regulating *MYB50* in the elongation zone. Next, we performed time-lapse imaging by using two fluorescent proteins. For time-lapse imaging, we fused estradiol-inducible *UPB1* with *CFP* (*pXVE*::*CFP-UPB1*) and transformed *pXVE*::*CFP-UPB1* into the *pMYB50*::*gMYB50-GFP/upb1-1* reporter line. Because GFP and CFP possess different emission wavelengths, we could detect the fluorescence from GFP and CFP simultaneously (S1 Movie). Transcription factors like UPB1 and MYB50 are usually localized in the nucleus. To minimize artifacts arising from nuclear movement, we quantified fluorescence from z-stacks containing 50 slices, choosing the focal plane with the sharpest-focused nucleus for each time point. Both CFP-cUPB1 and gMYB50-GFP fluorescence intensities were quantified in the early differentiation zone where root hair development initiated in the epidermis [21], and the fluorescence of MYB50-GFP clearly appeared (Fig 2B, 2C and S2 Fig). After 80 min of 5 μM estradiol treatment, CFP-UPB1 started to appear strong and GFP fluorescence began to decline (Fig 2B, 2C and S2 Movie). The CFP fluorescence intensity increased to approximately 5 times its initial time point level by 300 min after estradiol treatment, whereas GFP fluorescence continued to decrease to approximately 40% of its initial level by 200 min after estradiol treatment (Fig 2B, 2C and S2 Movie). In the absence of estradiol treatment, GFP fluorescence decreased to approximately 75% of its initial time point level by 300 min of imaging, while CFP fluorescence remained nearly unchanged and undetectable (Fig 2C). These findings indicate that the decrease in GFP fluorescence during estradiol treatment is significantly more pronounced than the decrease due to imaging, confirming that UPB1 represses *MYB50* expression upon estradiol treatment. Supporting this result, the mRNA levels of *UPB1* and *MYB50* in this multi-color reporter line were significantly altered after 5 h of estradiol treatment, similar to the fluorescence intensity (S3 Fig). These results indicate that UPB1 represses *MYB50* expression in the early differentiation zone.

### MYB50 regulates the length of the meristematic zone and mature cell length

**Fig 3:**
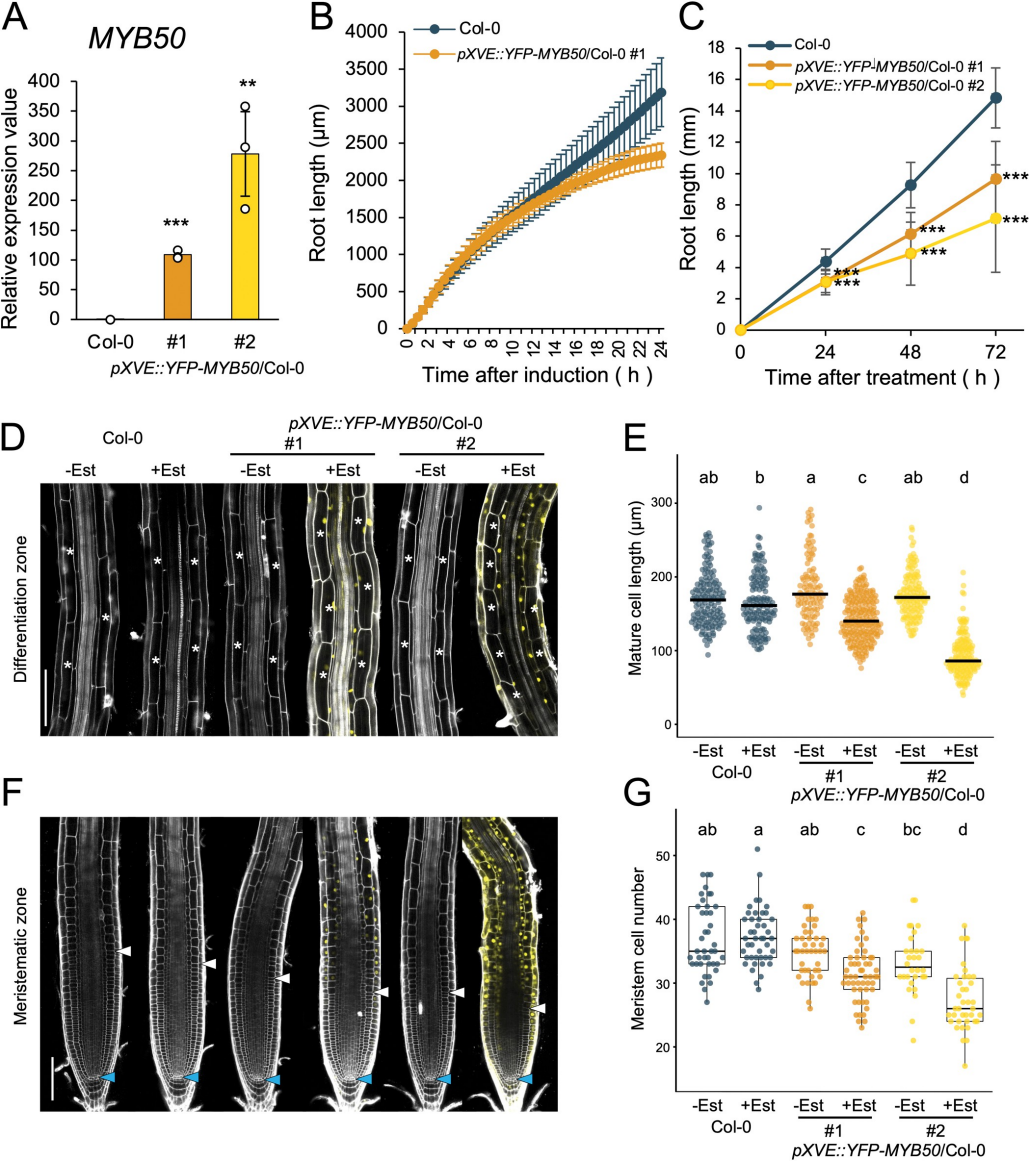
Root phenotype of *MYB50* induced-overexpressor (*pXVE*::*YFP-MYB50*). (A) *MYB50* expression after 24 h treatment of 6-day-old seedlings with 5 μM estradiol as measured by RT-qPCR (n = 3, mean ± SD). Significant differences were determined using Student’s *t*-test compared to Columbia (*** *p* < 0.001; ** *p* < 0.01). *p*-value: comparison of Columbia and #1, *p* < 0.001; comparison of Columbia and #2, *p* = 0.0052. (B) Root length increase in 4-day-old seedlings of Columbia (Col-0) and *pXVE*::*YFP-MYB50*/Columbia #1 treated with 5 μM estradiol within the agar medium. Root length was measured every 30 min using time-lapse imaging (n = 5, means ± SD). (C) Root length in 4-day-old seedlings grown vertically on agar medium containing 5 μM estradiol. Root length was measured every 24 h for 72 h (Columbia, n = 38; *pXVE*::*YFP-MYB50* #1, n = 39; *pXVE*::*YFP-MYB50* #2, n = 25; means ± SD). Statistically significant differences were determined using Student’s *t*-test by comparing Columbia and transgenic at each time (*** *p* < 0.001). (D) and (F) Confocal microscope images of 6-day-old roots treated with 5 μM estradiol (Est) for 24 h or untreated. Roots stained with propidium iodide. (D) The differentiation zone at a distance of 1 to 2 mm from each root tip. White asterisks indicate cortex cells with cell length measured. Scale bar, 100 μm. (E) Length of mature cortex cells. The black bar indicates the median. Mature cell length quantified for 10 to 15 roots, with approximately 3 to 10 cells per root. Measured cell numbers: Columbia—estradiol = 149, Columbia + estradiol = 141, *pXVE*::*YFP-MYB50* #1—estradiol = 101, #1 + estradiol = 209, *pXVE*::*YFP-MYB50* #2—estradiol = 130 and #2 + estradiol = 150. Statistically significant differences between samples determined using the Tukey’s honestly significant difference test (*p* < 0.001). (F) As (D) but showing the meristem. White arrowheads indicate the apparent end of the meristematic zone. Blue arrowheads indicate quiescent center cells. Scale bar, 100 μm. (G) Cortex cell number in the meristematic zone of 6-day-old roots treated with 5 μM estradiol (Est) or untreated. Counted root numbers: Columbia—estradiol = 41, Columbia + estradiol = 44, *pXVE*::*YFP-MYB50* #1—estradiol = 42, #1 + estradiol = 53, *pXVE*::*YFP-MYB50* #2—estradiol = 30, #2 + estradiol = 38. Statistically significant differences between samples determined using the Tukey’s honestly significant difference test (*p* < 0.001).

After showing that *MYB50* expression is repressed by UPB1, we next investigated how *MYB50* is involved in root growth. We attempted to analyze the *MYB50* function in root growth using genetic methods but an *MYB50* T-DNA insertion line is not available from the ABRC seed stock center. To investigate the effects of *MYB50* overexpression on root growth, we made a line expressing YFP-MYB50 under the control of an estradiol-inducible promoter (*pXVE*::*YFP-MYB50*). In this inducible line, *MYB50* expression reached more than 100 times the Columbia level in both independent lines after 24 h of estradiol treatment (Fig 3A). We investigated the root growth phenotype caused by induced *MYB50* with time-lapse imaging every 30 min for 24 h. After 24 h of induction with 5 μM estradiol, root elongation of *pXVE*::*YFP-MYB50* was inhibited (Fig 3B). In addition to time-lapse imaging, we measured the root length of MYB50 overexpressors on the vertical plates containing 5 μM estradiol at 24, 48, and 72 h (Fig 3C). After 72 h of estradiol treatment, the root length increments of *MYB50* overexpressors decreased to approximately 40% of that of the Columbia.

To determine the detailed effects of *MYB50* on root growth, we counted the number of cortical cells in the meristematic zone and measured the mature cell length in *MYB50* induced-overexpressor. Two independent lines of *pXVE*::*YFP-MYB50* treated with 5 μM estradiol for 24 h showed reduced cortical cell numbers in the meristematic zone and shorter mature cell length (Fig 3D–3G). Taken together, these results suggest that *MYB50* overexpression negatively regulates meristematic zone size and mature cell length, similar to *UPB1* overexpression. Because *UPB1* is known to control ROS homeostasis at the root tip, we investigated whether *MYB50* expression is induced by ROS. A 24 h treatment with H_2_O_2_ did not affect *MYB50* expression in wild type roots (S4A Fig). In addition to this, we investigated how the inhibition of root elongation under *MYB50* over-expression interacted with ROS-controlled root growth. H_2_O_2_, a ROS, is known to inhibit root elongation [22]. A combined treatment with estradiol and 500 μM H_2_O_2_ resulted in a smaller meristem size than estradiol treatment alone in Columbia roots (S4B and S4C Fig). Interestingly, *MYB50* induced-overexpressor showed smaller meristem sizes than Columbia plants in the estradiol treatment but even smaller meristem sizes upon H_2_O_2_ and estradiol treatment (S4B and S4C Fig). This additive effect suggests that the MYB50 could inhibit root growth independent of ROS regulation.

### Identification of MYB50-regulated genes

**Fig 4:**
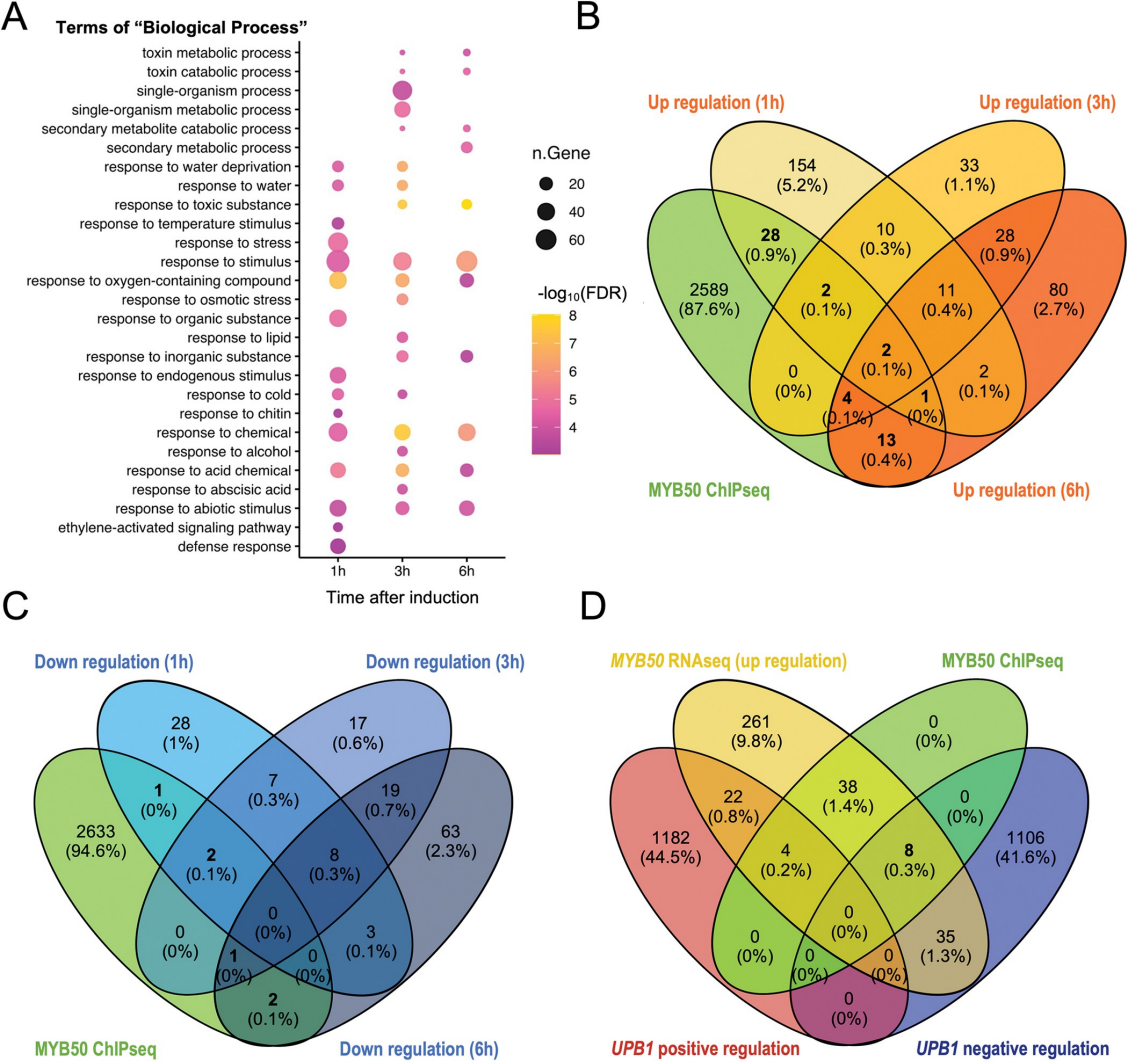
MYB50 regulated genes. (A) Biological processes in gene ontology (GO) enrichment analysis of upregulated genes in the *MYB50* induced-overexpressor (FDR < 0.001). The color and size of each point represents the -log_10_ (FDR) value and the number of genes, respectively. (B) Common genes between upregulated genes in each timepoint of RNA-seq and MYB50-bound genes by ChIP-seq. (C) Common genes between downregulated genes in each timepoint of RNA-seq and MYB50-bound genes by ChIP-seq. (D) Common genes between MYB50- and UPB1-regulated genes. *MYB50* RNA-seq (upregulation): Upregulated genes (368 genes) in at least a one-time point of estradiol induction of *pXVE*::*YFP-MYB50* in RNA-seq. MYB50 ChIP-seq: Common genes (50 genes) between upregulated genes by MYB50 and MYB50-bound genes in *pXVE*::*YFP-MYB50*. UPB1 positive regulation: Positively regulated genes (1,208) by UPB1. UPB1 negative regulation: Negatively regulated genes (1,149) by UPB1. UPB1 data were retrieved from Tsukagoshi et al., 2010 [7].

To identify the genes influenced by MYB50, we conducted RNA-seq and ChIP-seq analyses. To minimize the contribution from secondary and indirect relationships, we focused on genes induced by relatively short treatment with estradiol. We performed time-course RNA-seq analysis of *pXVE*::*YFP-MYB50* roots treated with 5 μM estradiol for 1, 3, and 6 h. Based on these analyses, we found that 210, 90, and 141 genes (singletons) were significantly upregulated at 1, 3, and 6 h, respectively (two-fold change, FDR < 0.001; S2 Table). In contrast, 49, 54, and 96 genes (singletons) were downregulated at 1, 3, and 6 h, respectively (two-fold change, FDR < 0.001; S3 Table). We analyzed the gene ontology (GO) terms of the “Biological Process” of upregulated genes in *MYB50*-induction lines and found that 15, 19, and 11 GO terms were significantly enriched at 1, 3, and 6 h, respectively (FDR < 0.001; Fig 4A). Among enriched GO terms, “response to acid chemical,” “response to abiotic stimulus,” “response to chemical,” and “response to oxygen-containing compounds,” were highly enriched. For the down-regulated genes, 6, 14, and 8 GO terms were significantly enriched at 1, 3, and 6 h, respectively (FDR < 0.001; S5 Fig). Among enriched GO terms, “response to stimulus”, “response to chemical”, “response to abiotic stimulus”, and “single-organism metabolic process” were highly enriched.

To identify MYB50 direct targets, we performed ChIP-seq analysis using 24 h estradiol-treated *pXVE*::*YFP-MYB50*. Sequence reads were mapped to the *A*. *thaliana* genome TAIR10 (https://www.arabidopsis.org/index.jsp) and genomic regions bound by *MYB50* according to an FDR *q*-value of < 10^−20^ in the dataset (S1 Dataset). ChIP-seq analysis identified 2,639 regions 1,000 bp upstream of the translation initiation site of genes. Of course, this number included false-positive genes because of the technical limitations of the ChIP-seq protocol. To find *MYB50*-biological direct target genes, we combined RNA-seq and ChIP-seq data. We searched for common genes between MYB50-bound genes in ChIP-seq and total genes that were upregulated (368 genes) or downregulated (151 genes) at least one time point during estradiol induction of *MYB50* in time-course RNA-seq. A statistically significant relationship was observed between 50 common genes and MYB50 bound genes (hypergeometric probability test, *p* = 3.23e^-07^), conversely, no such relationship was observed between six common genes of the downregulated genes and MYB50 bound genes (hypergeometric probability test, *p* = 0.151; Fig 4B and 4C). This suggests that MYB50 preferentially upregulates genes bound to MYB50 in the promoter region.

As *MYB50* was selected among UPB1 direct target genes, we compared differentially expressed genes (DEGs) between *UPB1* and *MYB50*-regulated genes (Fig 4D; [7]). Among the 368 genes upregulated in *pXVE*::*YFP-MYB50*, 43 were negatively regulated by UPB1 (1,149 genes are negatively regulated by UPB1: data retrieved from [7]). Eight genes were MYB50 ChIP-positive. In contrast, 26 genes were positively regulated by UPB1 (1,208 genes are positively regulated by UPB1: data retrieved from [7]). Four genes were positive for MYB50 by ChIP (Fig 4D and S4 Table). The number of common MYB50- and UPB1-regulated genes was not very high. The eight genes that were direct targets of MYB50 and were negatively regulated by UPB1 included stress-and ABA-responsible genes, such as *Dehydrin family protein* (*ERD10*; *At1g20450*), *CBL-interacting protein kinase 9* (*CIPK9*; *At1g01140*), and *cold-regulated 47* (*COR47*; *At1g20440*), and cell wall modification genes, such as *plant invertase/pectin methylesterase inhibitor superfamily protein* (*PMEI8*; *At3g17130*). Among these eight genes, *PMEI8* was the most strongly upregulated in the RNA-seq data sets (S6 Fig).

### MYB50 regulates the expression of pectin modification genes

**Fig 5:**
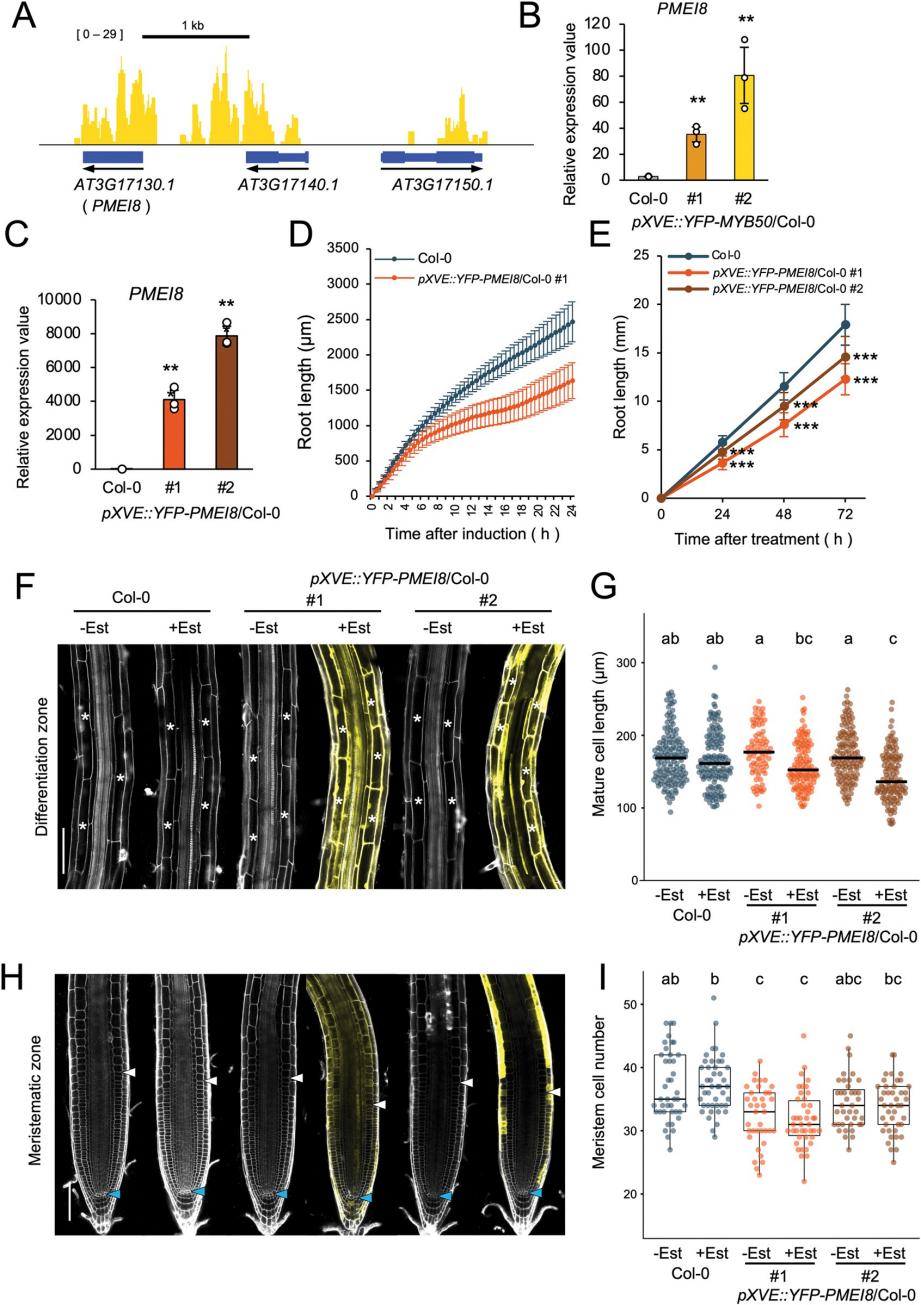
Root phenotype of *PMEI8* induced-overexpressor (*pXVE*::*YFP-PMEI8*). (A) MYB50 bound to the *PMEI8* promoter region in ChIP-seq. “Blue boxes” indicate ORFs (thick box: exon; thin box: intron) and black arrows beneath each ORF represents the direction of transcription. (B) *PMEI8* expression in 7-day-old roots after 24 h of 5 μM estradiol treatment as measured using RT-qPCR (n = 3, mean ± SD). Statistically significant differences were determined using Student’s *t*-test compared to Columbia (** *p* < 0.01). *p* value: comparison of Columbia and *pXVE*::*YFP-MYB50* #1, *p* = 0.00131; comparison of Columbia and *pXVE*::*YFP-MYB50* #2, *p* = 0.00702. (C) *PMEI8* expression in 6-day-old roots after 24 h of 5 μM estradiol treatment as measured using RT-qPCR (n = 3, mean ± SD). Statistically significant difference was determined using Student’s *t*-test compared to Columbia (*** *p* < 0.001). (D) Root length in 4-day-old seedlings treated with 5 μM estradiol within the agar medium. Root length was measured every 30 min for 24 h using time-lapse imaging (n = 5, means ± SD). (E) Root length in 4-day-old seedlings grown vertically on agar medium containing 5 μM estradiol. Root length was measured every 24 h for 72 h (Columbia, n = 53; *pXVE*::*YFP-PMEI8* #1, n = 33; *pXVE*::*YFP-PMEI8* #2, n = 32; means ± SD). Statistically significant differences were determined using Student’s *t*-test by comparing Columbia and *pXVE*::*YFP-PMEI8* at each time (*** *p* < 0.001). (F) and (H) Confocal microscope images of 6-day-old seedlings treated with 5 μM estradiol (Est) for 24 h or untreated. Roots were stained with propidium iodide (PI). (F) The differentiation zone is at a distance of 1 to 2 mm from each root tip. White asterisks indicate cortex cells with cell length measured. Scale bar, 100 μm. (G) Length of mature cortex cells in 7-day-old seedlings. Mature cell length was quantified for 10–15 roots, with approximately 3–10 cells per root. The black bar indicates the median. Measured cell numbers: Columbia -estradiol = 149; Columbia +estradiol = 141; *pXVE*::*YFP-PMEI8* #1 -estradiol = 81; #1 +estradiol = 120; *pXVE*::*YFP-PMEI8* #2 -estradiol = 131; #2 +estradiol = 119. Statistically significant differences between samples determined using the Tukey’s honestly significant difference test (*** *p* < 0.001). (H) As (F) but showing the meristem. White arrowheads indicate the apparent end of the meristematic zone. Blue arrowheads indicate the quiescent center cells (QC). Scale bar, 100 μm. (I) Cortex cell number in the meristematic zone of 6-day-old seedlings treated with 5 μM estradiol (Est) or untreated. Counted root numbers: Columbia -estradiol = 41; Columbia +estradiol = 44; *pXVE*::*YFP-PMEI8* #1 -estradiol = 37; #1 +estradiol = 42; *pXVE*::*YFP-PMEI8* #2 -estradiol = 39; #2 +estradiol = 42. Statistically significant differences between samples determined using the Tukey’s honestly significant difference test (*** *p* < 0.001).

Among the *MYB50* direct target genes previously identified as differentially expressed in *upb1-1* [7], we focused on *PMEI8* (*At3g17130*) and its role in root growth. PMEIs are known to regulate pectin status, and *PMEI8* overexpression partially complements the short-root phenotype in *cobra* mutants [23]. However, the detailed effects of *PMEI8* overexpression on root growth have not been reported. *In silico* analysis of RootMap gene expression data [24] showed that *PMEI8* is highly expressed in sections 7 to 9 (from the elongation zone close to the early differentiation zone) (S7 Fig). Moreover, the expression of MYB50 in the RootMap dataset peaks at section 7, consistent with the idea that MYB50 regulates *PMEI8* expression in the elongation and early differentiation zones (Fig 2A). In addition, MYB50 bound approximately 1,000 bp upstream of the *PMEI8* coding region according to our ChIP-seq data (Fig 5A). As *PMEI8* was upregulated in the *MYB50* induced-overexpressor (Fig 5B), we decided to investigate the phenotype of an overexpressor of *PMEI8*.

To investigate how *PMEI8* overexpression affected root growth, we made a *YFP-PMEI8* inducible line (*pXVE*::*YFP-PMEI8*) that expressed *PMEI8* more than 4,000 times after 24 h of estradiol treatment (Fig 5C). Time-lapse imaging was performed every 30 min after 5 μM estradiol treatment to measure root elongation. After 24 h estradiol treatment, elongation of *pXVE*::*YFP-PMEI8* was approximately 70% compared to Columbia (Fig 5D). We also measured the root length of *PMEI8* overexpressors on the vertical plates containing 5 μM estradiol every 24 h for a duration of 72 h. After 48 and 72 h of induction with estradiol, root lengths of *PMEI8* overexpressors were slightly shortened compared to Columbia (Fig 5E). We measured the number of cortical cells in the meristematic zone and the mature cell length in the *PMEI8* induced-overexpressors. Both the cell numbers in the meristematic zone of no-estradiol and estradiol-treated *pXVE*::*YFP-PMEI8* plants were slightly reduced compared to those of Columbia (#1 was statistically significant but #2 was not statistically significant). Estradiol-treated *pXVE*::*YFP-PMEI8* plants showed significantly shorter mature cell length than control cells (Fig 5F–5I). The mature cell length phenotype was consistent with *MYB50* induced-overexpressor in which MYB50 directly upregulated *PMEI8* expression (Fig 5A and 5B). This result indicates that *PMEI8* is a negative regulator of root growth, especially cell length, under the MYB50 transcriptional regulation.

## Discussion

### *MYB50* is a direct downstream target of UPB1

In a previous study, UPB1 was shown to regulate root development through reactive oxygen species (ROS) homeostasis at the root tip by directly controlling the expression of peroxidase genes [7]. In the study, UPB1 is shown to bind to the promoter regions of several transcription factor genes that are probably not involved in ROS homeostasis. We hypothesized that UPB1-targeted transcription factors also regulate root growth through a regulatory network independent of ROS. Among these, we were interested in a transcription factor whose function has not yet been reported. In this study, we selected *MYB50* as a candidate gene that is part of the UPB1 regulatory network for root growth. *MYB50* expression was upregulated in the *upb1-1* mutant but downregulated in the *UPB1* overexpressor. As *MYB50* is expressed in the elongation zone, *MYB50* is likely to have a role in the root development. Similarly, an *MYB50* induced-overexpressor showed decreased root elongation compared to the control. These results indicate that *MYB50* is a root growth inhibitor. We confirmed *UPB1*-*MYB50* transcriptional regulation by multi-color reporter lines. Visualizing transcriptional regulation is challenging; however, recent imaging technologies have allowed us to achieve this goal. MYB50-GFP expression was repressed after the estradiol induction of CFP-UPB1 in root cells. This system might be used not only for other gene regulatory networks of transcription factors but also for tracing alterations in the subcellular localization of several proteins simultaneously.

### MYB50 regulated *PMEI8* expression under the UPB1 gene regulatory network

Transcriptome analysis of *MYB50* induction indicated that MYB50 did not regulate the expression of ROS homeostasis genes regulated by UPB1. In support of this, H_2_O_2_ treatment had an additive effect on the reduction in meristem size in the *MYB50* overexpressor. Interestingly, the number of common MYB50 and UPB1-regulated genes was low. As the *MYB50* expression level by the *pXVE* promoter was much stronger than that in *upb1-1* mutants, *MYB50* overexpression affected its downstream genes more than the UPB1 gene regulatory network. However, we found one interesting gene, *PMEI8*, among *MYB50* target genes which was also derepressed in the *upb1-1* mutant. Our results showed that *PMEI8* under the MYB50 gene regulatory network inhibited cell elongation and consequently shortened mature cell length.

Fine-tuning of pectin methyl esterification is considered important for cell wall stiffening, which regulates cell growth [25, 26]. Spatiotemporal control of pectin methyl esterification is affected by PMEs and PMEIs in *A*. *thaliana*. Although PMEI family proteins are predicted to have the same function, each PMEI, when knocked out or over-expressed, can give rise to different root phenotypes. *PMEI-1*, *PMEI-2*, or *PMEI9* overexpression resulted in a longer primary root phenotype, whereas *PMEI4* overexpression resulted in a short root phenotype [27, 28]. These results on PMEI function support the hypothesis that spatiotemporally controlled PMEIs regulate root growth. Moreover, overexpression of another *PMEI13* (*At5g62360*) did not alter root length; however, overexpression of that *PMEI13* with *EXPA5*, which is considered a cell wall-loosening enzyme, increased root length [29].

*PMEI8* has been identified as a partial suppressor of *cobra* mutations. *PMEI8* overexpression in *cobra* mutants can ameliorate their root growth defects [23]. According to these functions, there are coregulatory genes of PMEI8 in the *MYB50* and *UPB1* regulatory gene networks.

**Fig 6:**
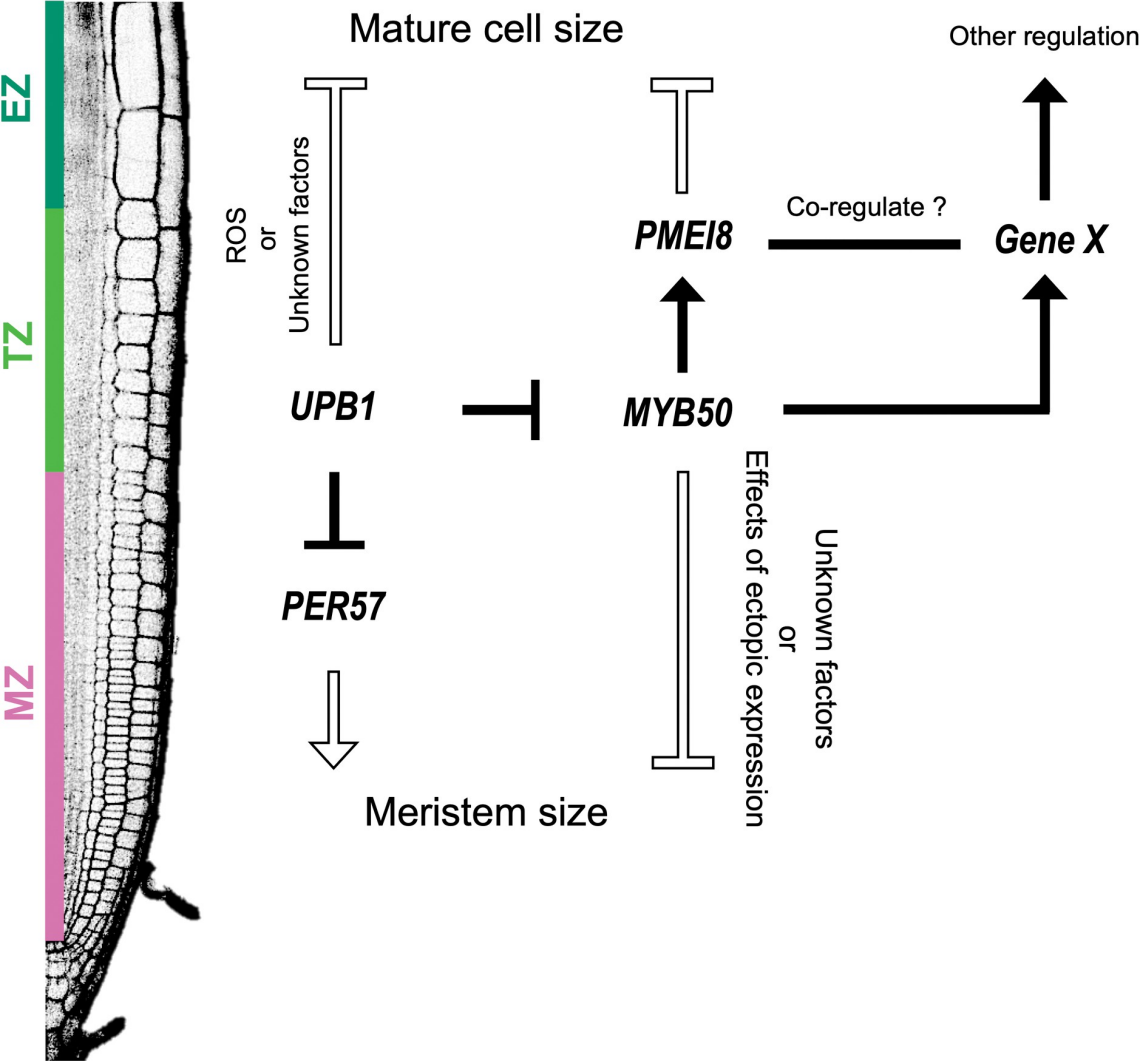
A schematic model of gene regulatory network under UPB1 and MYB50 control. Black arrows and “T”s indicate transcriptionally direct target genes. The outline arrow and “T”s indicate an effect on the root phenotype. (MZ): Meristematic zone. (TZ): Transition zone. (EZ): Elongation zone. UPB1 directly represses *PER57* in the TZ and negatively regulates the meristem size [7]. In the EZ, UPB1 directly represses *MYB50* expression. The root phenotypic analysis using *pXVE*::*YFP-MYB50* revealed that MYB50 negatively regulates the meristem size through a pathway separate from ROS regulation, whereas the cause remains unknown. However, *MYB50* was expressed in the EZ close to the differentiation zone and directly activated *PMEI8* expression. *MYB50* regulatory network and *PMEI8* inhibit cell elongation as *pXVE*::*YFP-MYB50* and *pXVE*::*YFP-PMEI8* shortened mature cell length. The MYB50-regulated transcriptional network includes several cell wall-associated genes, which may coregulate with *PMEI8* to exert some coregulation.

The data presented here, combined with previous *UPB1* data, are shown as a model of the gene regulatory network (Fig 6). UPB1 negatively impacts meristem size through ROS homeostasis by repressing several peroxidase genes, which are associated with larger meristems when overexpressed [7]. In our study, we demonstrated that UPB1 directly represses *MYB50* in the elongation zone. MYB50, in turn, negatively regulates meristem size, with the *MYB50-*induced overexpressor displaying smaller meristem size and mature cell length (Fig 3E and 3F).

The mechanism behind MYB50’s suppression of meristem size remains unclear. Concerning cell elongation, we found that MYB50 suppresses it through the transcriptional activation of downstream genes, one of which is *PMEI8*. In summary, the integration of both UPB1 and MYB50 transcriptional networks indicates that UPB1 not only controls ROS homeostasis but also influences cell wall status, particularly pectin modification via *PMEI8*, a direct target of MYB50. However, the regulation of PMEI8 alone does not entirely elucidate the complex regulatory mechanisms of root growth within the MYB50 network. To support this hypothesis, we identified several cell wall modification genes in the transcriptome datasets. To fully understand the *MYB50* gene regulatory network that controls root growth, we need to investigate the gene functions in the near future.

We found that 368 genes were significantly upregulated in the RNA-seq analysis of the *MYB50* induction line. Among the upregulated genes, we identified GO terms representing the responses to abiotic stress and chemical compounds (Fig 4A). Furthermore, we combined these data with ChIP-seq data from YFP-MYB50, concluded that *MYB50* was a transcriptional activator, and identified 50 genes as MYB50 direct target genes. We identified *ABA repressor 1* (*ABR1*), *NAC004*, *ERF019*, and *CBF2* [30, 31]. These transcription factors are all involved in the abscisic acid (ABA) response, especially in stress responses such as cold and drought. In addition to these transcription factors, we identified ABA and stress response marker genes such as *LEA*, *RD29A*, *ERD4*, *ERD10*, and *COR47* [32–38]. These results indicated that *MYB50* may be involved in plant stress responses regulated by ABA. In future studies, to deepen our understanding of the function of *MYB50* in ABA signaling, we need to investigate the phenotypes of *MYB50* overexpressor under abiotic stress.

## Conclusions

In this study, we identified a transcription factor, *MYB50*, that is one of downstream genes of UPB1 for regulating root growth. We demonstrated MYB50’s direct role in inducing the expression of *PMEI8*, which participates in cell wall modification and influences root growth. While UPB1 regulates root growth through maintaining reactive oxygen species (ROS) homeostasis at the root tip, our findings suggest that MYB50, by controlling *PMEI8* expression, also contributes to root growth regulation by influencing cell wall status. Root growth is complex, and the function of a single transcription factor alone is insufficient to provide a comprehensive explanation. The functional analysis of MYB50 underscores the intricate interplay between the ROS regulatory pathway and the cell wall modification system via PMEI8, regulated by MYB50 downstream of UPB1, which is vital for normal root development. However, *PMEI8* regulation alone does not fully elucidate the regulatory mechanisms of root growth in the MYB50 network. As we also found several transcription factors in the MYB50 direct target genes, further analysis of their functions will provide more comprehensive insights into the regulatory mechanisms of root growth within the MYB50 gene regulatory network.

## Supporting information

S1 Fig Expression of *pMYB50*::*gMYB50-GFP* in Columbia and *upb1-1* mutant background. Confocal microscopy image of 7-day-old roots of *pMYB50*::*gMYB50-GFP* in Columbia (A) and *upb1-1* mutant background (B) from Fig 2A. Scale bars, 100 μm.(C) Thirty Z-stack images in the meristematic, elongation, and early differentiation zones of (A). (D) Thirty Z-stack images in the meristematic, elongation, and early differentiation zones of (B). White arrow heads indicate the nuclei with apparent GFP fluorescence. Maximum projections were constructed from all 30 Z-stack images.
(TIF)

S2 Fig Quantification of *MYB50* repression by UPB1 at the single cell level using multi-color reporter line. Time-lapse images of z-stacks at the early differentiation zone of *pXVE*::*CFP-cUPB1*/*pMYB50*::*gMYB50-GFP/upb1-1* were taken by LAS X every 20 min for 5 h. Among 50 z-stacks for each time point, the image which captured the nucleus most clearly was selected because the nucleus moves rapidly in the cells during imaging. CFP and GFP fluorescence of each nuclear signal in the selected images were quantified as a mean intensity by Fiji software. “Z” indicates the z-stack number. “T” indicates the time point after starting time-lapse imaging.
(TIF)

S3 Fig *UPB1* and *MYB50* expression in the multicolor reporter line. *UPB1* and *MYB50* expression in *pXVE*::*CFP-cUPB1*/*pMYB50*::*gMYB50-GFP*/*upb1-1* after 5 h of 5 μM estradiol (Est) treatment as measured by RT-qPCR (n = 5, mean ± SD). Statistically significant differences were determined using Student’s *t*-test and compared with untreated plants (*** *p* < 0.001; * *p* < 0.05). *p* value: *UPB1* expression, *p* < 0.001; *MYB50* expression, *p* = 0.0168.
(TIF)

S4 Fig Effects of H_2_O_2_ in *MYB50* induced-overexpressor. (A) *MYB50* expression level in the root of Columbia with or without 500 μM H_2_O_2_ treatment for 24 h (n = 3). Bars show mean ± SD. (B) Confocal images of the meristematic zone treated with 5 μM estradiol (Est) or both 5 μM estradiol and 500 μM H_2_O_2_ treatment for 24 h. Roots were stained with propidium iodide (PI). White arrowheads indicate the ends of the meristematic zone. The blue arrowheads indicate quiescent center cells. Scale bar, 100 μm. (C) Cortex cell number in the meristematic zone of 6-days-old seedlings with 5 μM estradiol (Est) or both 5 μM estradiol and 500 μM H_2_O_2_ (Est + H_2_O_2_) for 24 h. Counted root numbers: Columbia estradiol = 44; Columbia estradiol+ H_2_O_2_ = 38; *MYB50* induced-overexpressor #1 estradiol = 53; #1 estradiol+ H_2_O_2_ = 46; #2 estradiol = 38, and #2 estradiol+ H_2_O_2_ = 37. Statistically significant differences were determined using Student’s *t*-test compared to estradiol alone and both 5 μM estradiol and 500 μM H_2_O_2_ treatment (*** *p* < 0.001).
(TIF)

S5 Fig Biological processes in gene ontology (GO) enrichment analysis of downregulated genes in *MYB50* induced-overexpressor. GO enrichment was cut off by FDR < 0.001. The color and size of each point represented the -log_10_ (FDR) values and the number of genes, respectively.
(TIF)

S6 Fig Expression of eight direct target genes of MYB50 negatively regulated by UPB1. Expression patterns of selected eight genes from the integration of MYB50 and UPB1 networks in a time-course RNAseq analysis of pXVE::YFP-MYB50/Columbia roots treated with 5 μM estradiol for 1, 3, or 6 h. The expression values at each time point are relative to the value at 0 h from the RNA-seq results. Error bars indicate SE; (* q-value < 0.05; ** q-value < 0.01; *** q-value < 0.001). p value: ERD10 expression after 3 h, p = 0.0247; COR47 expression after 3 h, p = 0.0045.
(TIF)

S7 Fig *MYB50* and *PMEI8* expressions in the root. *MYB50* and *PMEI8* expression data from root map longitudinal datasets [24]. Section 1–6 correspond to the meristematic zone. Section 7 and 8 correspond to the elongation zone. Section 9–12 describe the differentiation zones.
(TIF)

S1 Table Primers used in this study. (XLSX)

S2 Table Significantly upregulated genes in the time-course RNA-seq used *MYB50* induced-overexpressor. (XLSX)

S3 Table Significantly downregulated genes in the time-course RNA-seq used *MYB50* induced-overexpressor. (XLSX)

S4 Table List of overlapping genes between MYB50- and UPB1-regulated genes, related to Fig 4D. (XLSX)

S1 Dataset List of genes in which MYB50 bound 1,000 bp upstream in the ChIP-seq. (XLSX)

S2 Dataset Minimal data set. (XLSX)

S1 Movie Time-lapse imaging (tile-scan) of *pXVE*::*CFP-cUPB1*/*pMYB50*::*gMYB50-GFP*/*upb1-1*. 6-day-old seedlings of *pXVE*::*CFP-cUPB1*/*pMYB50*::*gMYB50-GFP/upb1-1* were transferred from the MS medium to chambered cover glass in media containing 5 μM estradiol. The roots were then stained with PI. Time-lapse images were captured using LAS X every 40 min for 7 h and 20 min.
(AVI)

S2 Movie A time-lapse image of the nuclei of *pXVE*::*CFP-cUPB1*/*pMYB50*::*gMYB50-GFP*/*upb1-1* with measured fluorescence intensity. Representative time-lapse image of selected nuclei with measured fluorescence intensity for GFP and CFP. Each frame was selected from a set of 50 z-stacks where fluorescence was most sharply observed.
(AVI)
