# Supplementary figures and images for: AtMYB50 regulates root cell elongation by upregulating PECTIN METHYLESTERASE INHIBITOR 8 in Arabidopsis thaliana
Source: PLoS One. 2023 Dec 22;18(12):e0285241. doi: 10.1371/journal.pone.0285241 (PMC10745173; doi:10.1371/journal.pone.0285241)

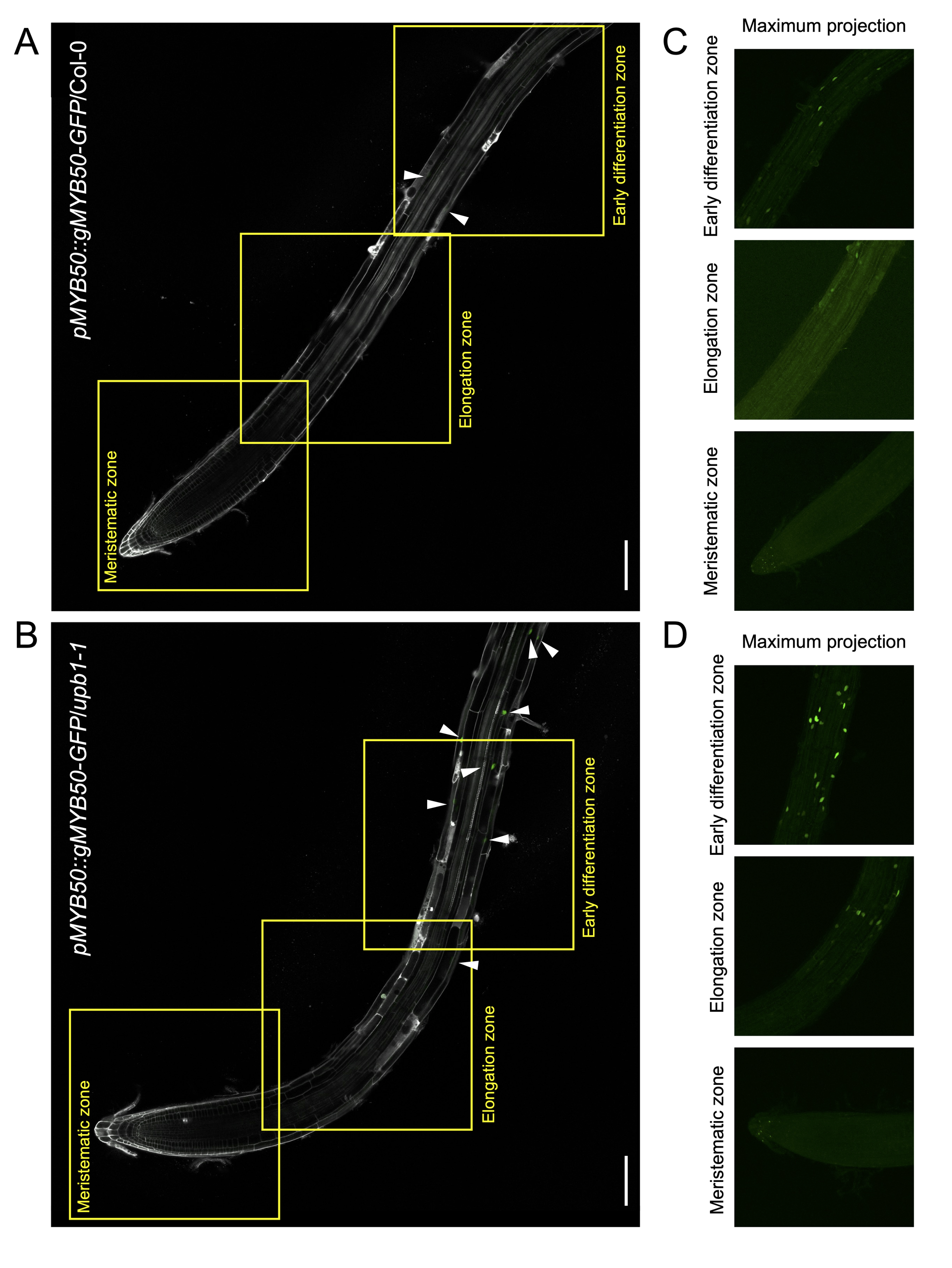

Supplement: S1 Fig — Confocal microscopy image of 7-day-old roots of pMYB50::gMYB50-GFP in Columbia (A) and upb1-1 mutant background (B) from Fig 2A. Scale bars, 100 μm.(C) Thirty Z-stack images in the meristematic, elongation, and early differentiation zones of (A). (D) Thirty Z-stack images in the meristematic, elongation, and early differentiation zones of (B). White arrow heads indicate the nuclei with apparent GFP fluorescence. Maximum projections were constructed from all 30 Z-stack images. (TIF) [file pone.0285241.s001.tif]

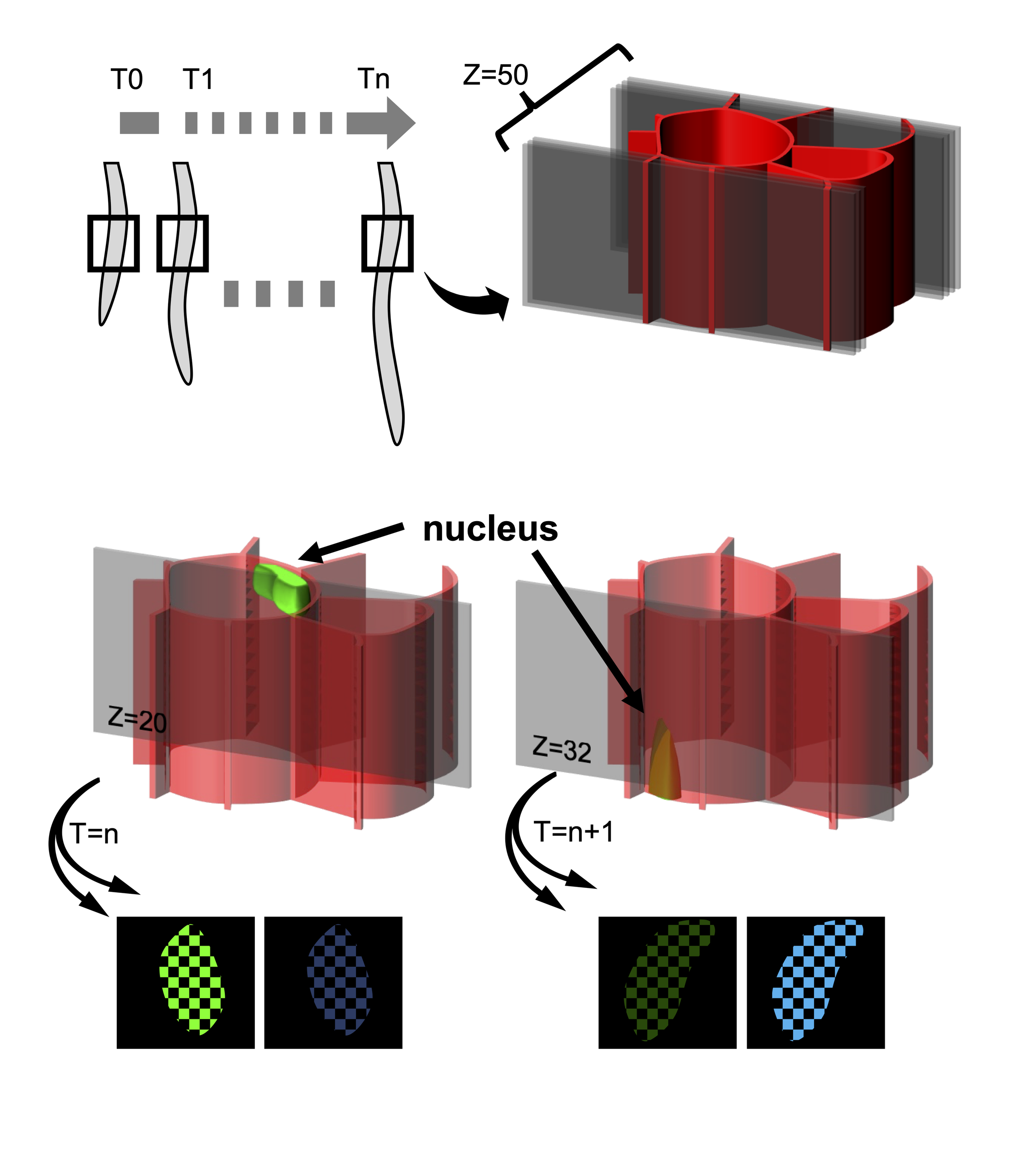

Supplement: S2 Fig — Time-lapse images of z-stacks at the early differentiation zone of pXVE::CFP-cUPB1/pMYB50::gMYB50-GFP/upb1-1 were taken by LAS X every 20 min for 5 h. Among 50 z-stacks for each time point, the image which captured the nucleus most clearly was selected because the nucleus moves rapidly in the cells during imaging. CFP and GFP fluorescence of each nuclear signal in the selected images were quantified as a mean intensity by Fiji software. “Z” indicates the z-stack number. “T” indicates the time point after starting time-lapse imaging. (TIF) [file pone.0285241.s002.tif]

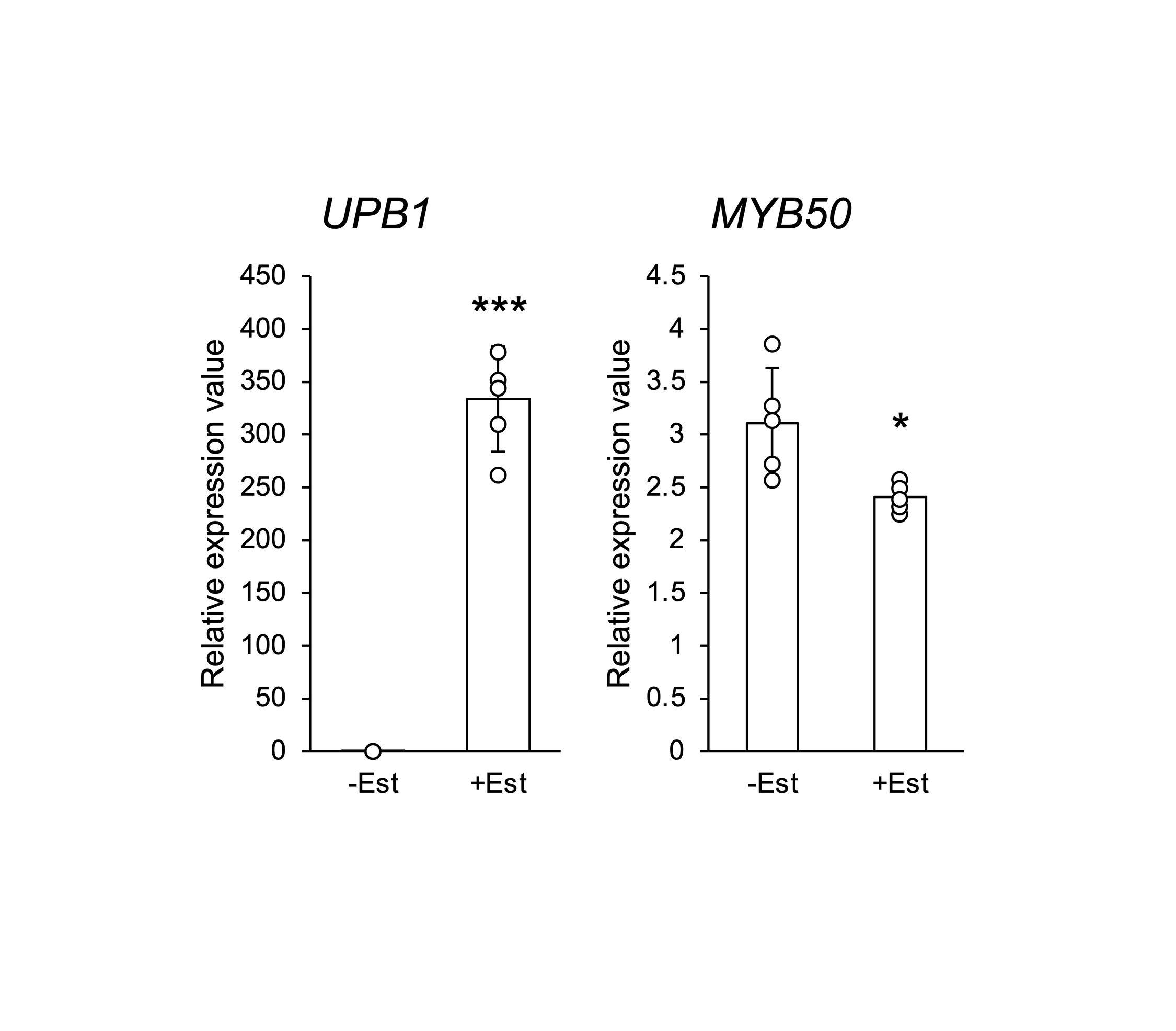

Supplement: S3 Fig — UPB1 and MYB50 expression in pXVE::CFP-cUPB1/pMYB50::gMYB50-GFP/upb1-1 after 5 h of 5 μM estradiol (Est) treatment as measured by RT-qPCR (n = 5, mean ± SD). Statistically significant differences were determined using Student’s t-test and compared with untreated plants (*** p < 0.001; * p < 0.05). p value: UPB1 expression, p < 0.001; MYB50 expression, p = 0.0168. (TIF) [file pone.0285241.s003.tif]

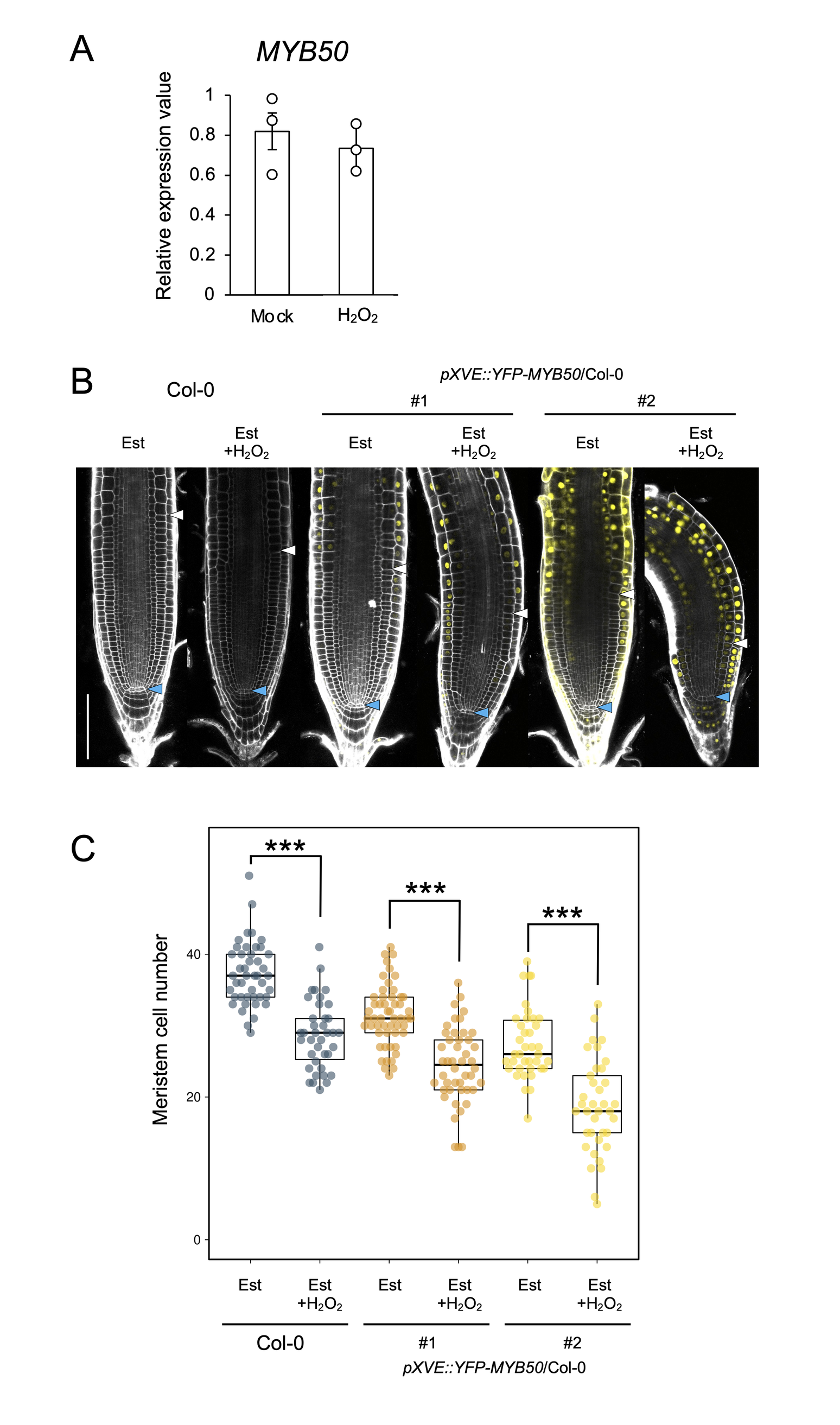

Supplement: S4 Fig — (A) MYB50 expression level in the root of Columbia with or without 500 μM H2O2 treatment for 24 h (n = 3). Bars show mean ± SD. (B) Confocal images of the meristematic zone treated with 5 μM estradiol (Est) or both 5 μM estradiol and 500 μM H2O2 treatment for 24 h. Roots were stained with propidium iodide (PI). White arrowheads indicate the ends of the meristematic zone. The blue arrowheads indicate quiescent center cells. Scale bar, 100 μm. (C) Cortex cell number in the meristematic zone of 6-days-old seedlings with 5 μM estradiol (Est) or both 5 μM estradiol and 500 μM H2O2 (Est + H2O2) for 24 h. Counted root numbers: Columbia estradiol = 44; Columbia estradiol+ H2O2 = 38; MYB50 induced-overexpressor #1 estradiol = 53; #1 estradiol+ H2O2 = 46; #2 estradiol = 38, and #2 estradiol+ H2O2 = 37. Statistically significant differences were determined using Student’s t-test compared to estradiol alone and both 5 μM estradiol and 500 μM H2O2 treatment (*** p < 0.001). (TIF) [file pone.0285241.s004.tif]

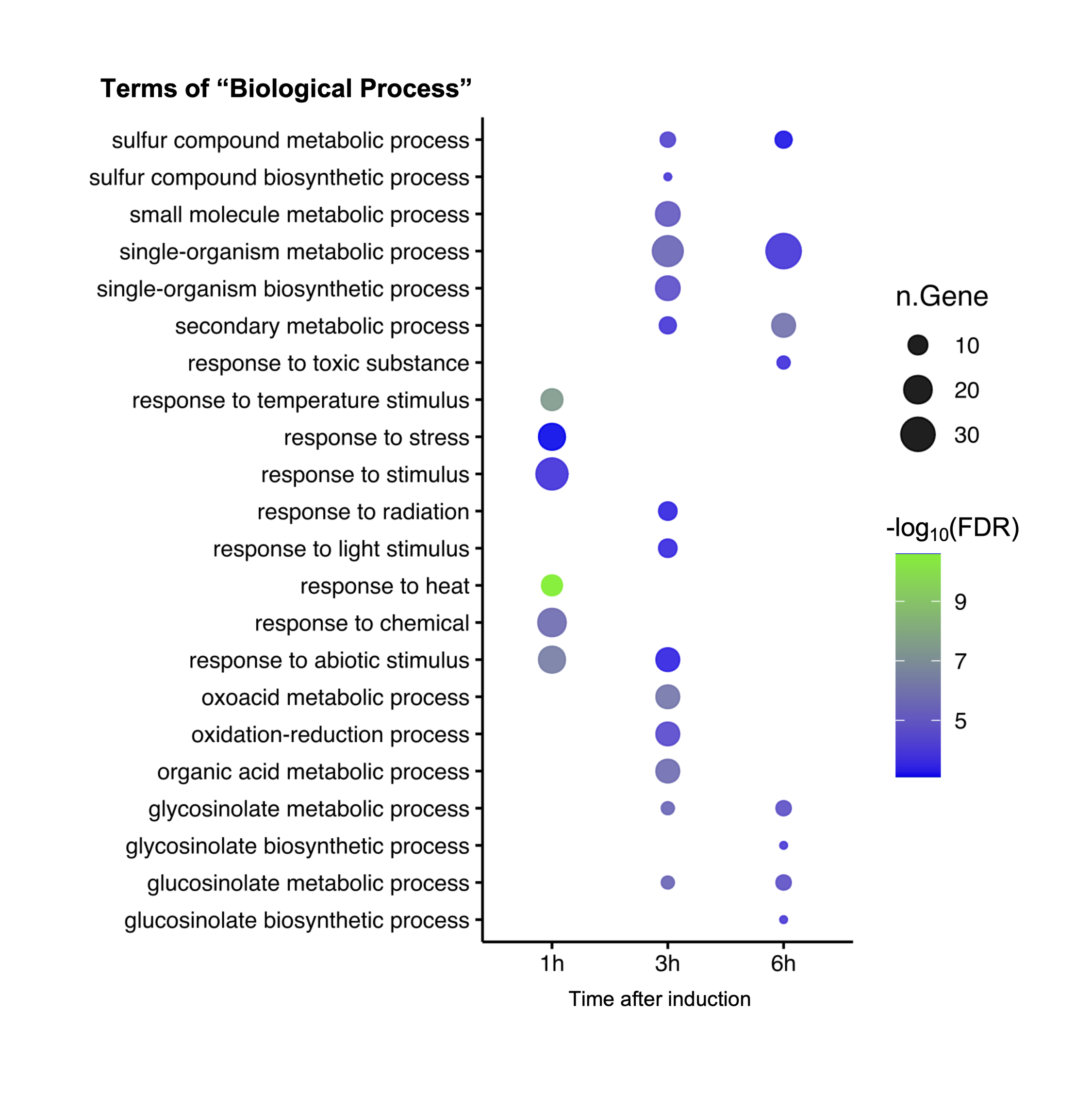

Supplement: S5 Fig — GO enrichment was cut off by FDR < 0.001. The color and size of each point represented the -log10 (FDR) values and the number of genes, respectively. (TIF) [file pone.0285241.s005.tif]

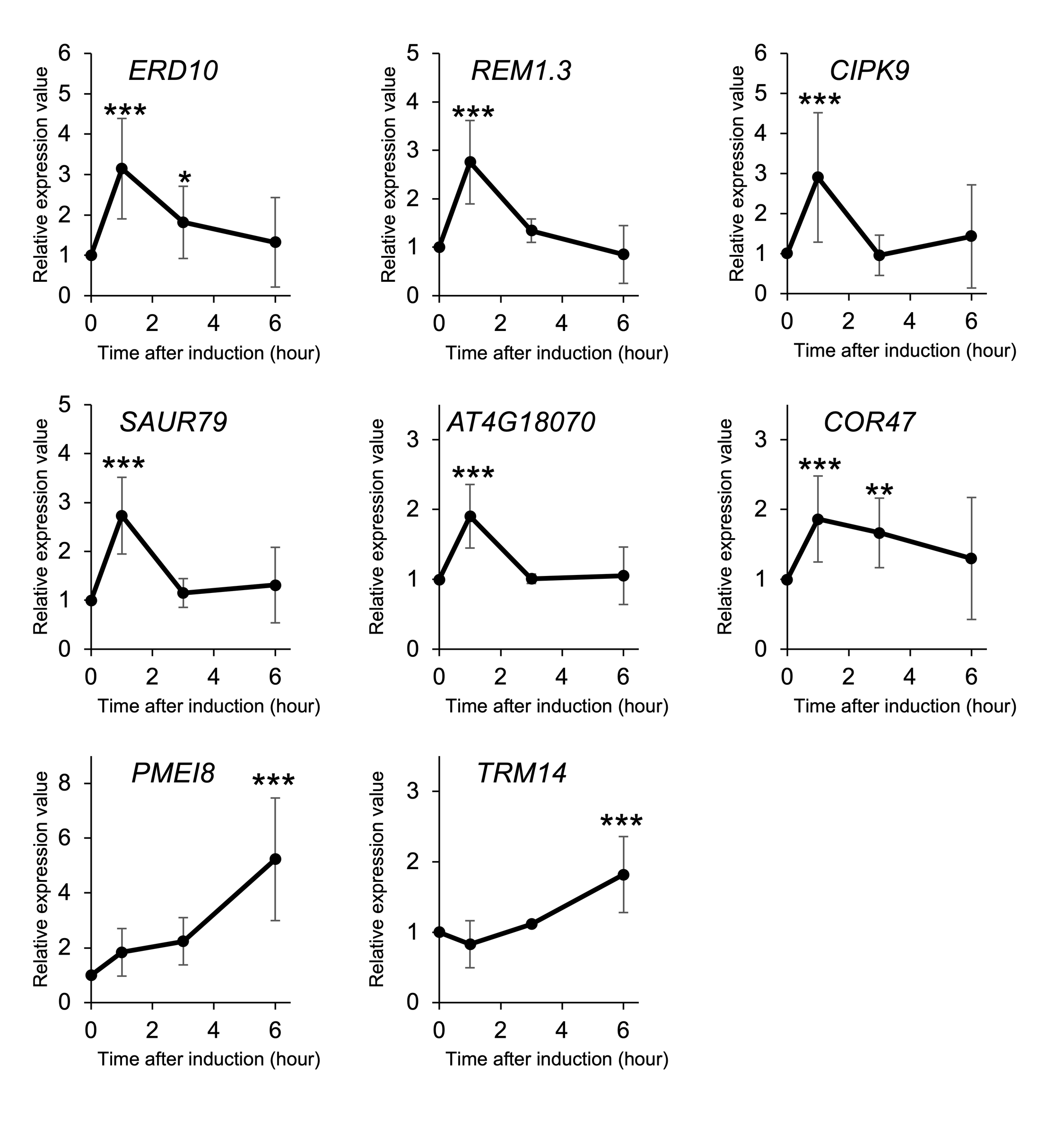

Supplement: S6 Fig — Expression of eight direct target genes of MYB50 negatively regulated by UPB1. Expression patterns of selected eight genes from the integration of MYB50 and UPB1 networks in a time-course RNAseq analysis of pXVE::YFP-MYB50/Columbia roots treated with 5 μM estradiol for 1, 3, or 6 h. The expression values at each time point are relative to the value at 0 h from the RNA-seq results. Error bars indicate SE; (* q-value < 0.05; ** q-value < 0.01; *** q-value < 0.001). p value: ERD10 expression after 3 h, p = 0.0247; COR47 expression after 3 h, p = 0.0045. (TIF) [file pone.0285241.s006.tif]

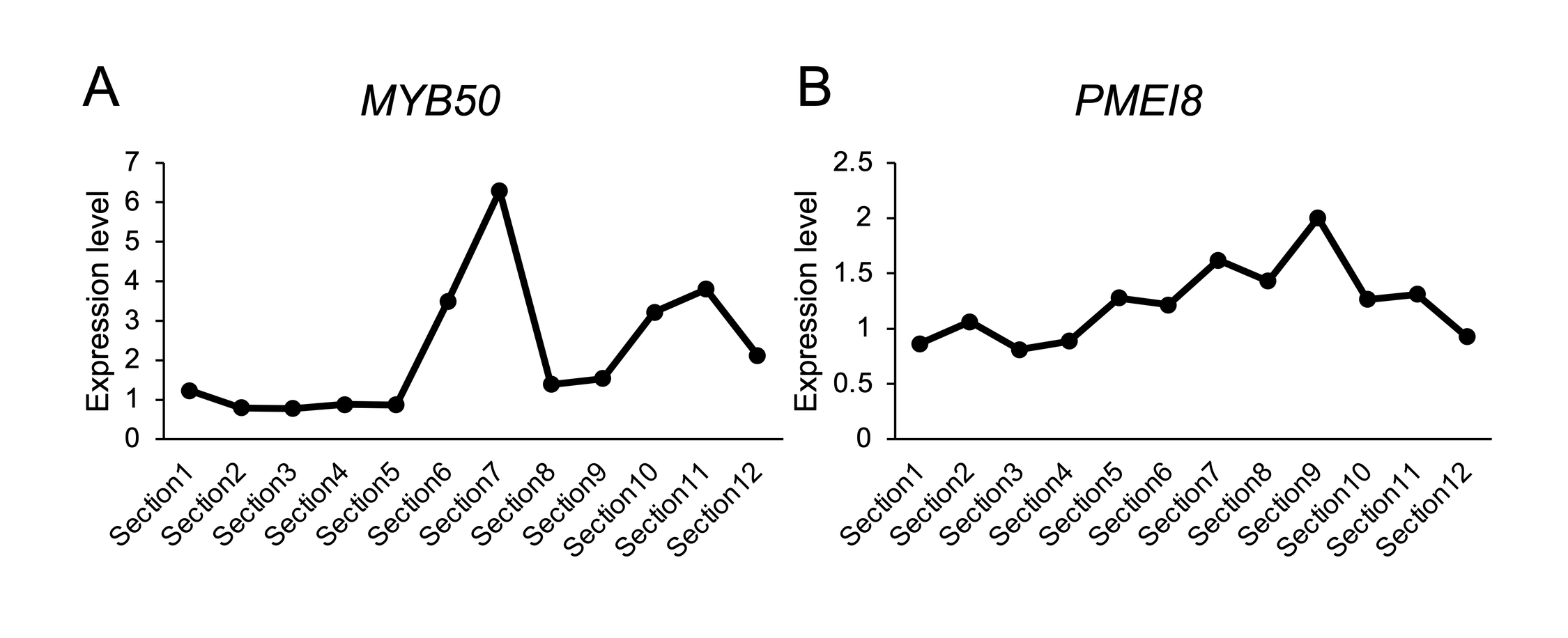

Supplement: S7 Fig — MYB50 and PMEI8 expression data from root map longitudinal datasets [24]. Section 1–6 correspond to the meristematic zone. Section 7 and 8 correspond to the elongation zone. Section 9–12 describe the differentiation zones. (TIF) [file pone.0285241.s007.tif]
